# Exonuclease resistant 18S and 25S ribosomal RNA components in yeast are possibly newly transcribed by RNA polymerase II

**DOI:** 10.1186/s12860-020-00303-z

**Published:** 2020-08-01

**Authors:** Jacob Fleischmann, Miguel A. Rocha, Peter V. Hauser, Bhavani S. Gowda, Mary Grace D. Pilapil

**Affiliations:** 1 Research Division, Greater Los Angeles VA Healthcare System, Los Angeles, California USA; 2 Department of Medicine, David Geffen School of Medicine at UCLA, Los Angeles, California USA; 3 Department of Integrative Biology and Physiology, University of California at Los Angeles, Los Angeles, California USA

## Abstract

**Background:**

We have previously reported 18S and 25S ribosomal RNA molecules in *Candida albicans* resistant to processive 5′ → 3′ exonuclease, appearing as cells approached stationary growth phase. Initial analysis pointed to extra phosphate(s) at their 5′- end raising the possibility that they were newly transcribed. Here we report on additional experiments exploring this possibility and try to establish which of the RNA polymerases may be transcribing them.

**Results:**

Oligo-ligation and primer extension again showed the presence of extra phosphate at the 5′-end of the reported processing sites for both 18S and 25S ribosomal RNA components. Inhibition of Pol I with BMH-21 increased the presence of the molecules. Quantitation with an Agilent Bioanalyzer showed that resistant 18S and 25S molecules are primarily produced in the nucleus. Utilizing an RNA cap specific antibody, a signal could be detected on these molecules via immunoblotting; such signal could be eliminated by decapping reaction. Both the cap specific antibody and eIF4E cap-binding protein, increased fold enrichment upon quantitative amplification. Antibodies specific for the RNA Polymerase II c-terminal domain and TFIIB initiator factor showed the presence of Pol II on DNA sequences for both 18S and 25S molecules in chromatin precipitation and qPCR assays. Rapamycin inhibition of TOR complex also resulted in an increase of resistant 18S and 25S molecules.

**Conclusions:**

These data raise the possibility of a role for RNA Polymerase II in the production of 18S and 25S molecules and indicate that efforts for more direct proof may be worthwhile. If definitively proven it will establish an additional role for RNA Polymerase II in ribosomal production.

## Background

Ribosome biogenesis in yeast, most extensively studied in *Saccharomyces cerevisiae,* requires a multistep process that includes ribosomal RNA (rRNA) transcription, pre-ribosomal RNA processing, ribosome assembly and export. While three RNA polymerases are involved in ribosome production, the 18S, 5.8S and 25S ribosomal RNA (rRNA) components are thought to be products of polycistronic transcription by RNA polymerase I (Pol I) followed by processing [1–3]. The fourth rRNA component 5S is transcribed in the reverse direction by Pol III [4, 5] and Pol II transcribes the genes coding for ribosome associated proteins [6]. A role for Pol II in ribosomal RNA production in *Saccharomyces cerevisiae* has been described [7]. When *RRN9*, one of the components of Pol I upstream activating factor (UAF) was deleted, inactivating Pol I, the yeast was capable of ribosome production utilizing Pol II. Transcription was initiated from multiple start sites upstream or downstream from the normal Pol I’s promoter site, still in a polycistronic fashion. It has also been seen in a petite strain of *S. cerevisiae,* involving the selective activation of cryptic Pol II promoters from episomal rDNA elements [8].

The polymorphic yeast *Candida albicans* is a major cause of invasive fungal disease, especially in immune compromised patients [9]. As in *Saccharomyces cerevisiae*, genes coding for rRNA (rDNA) in *C. albicans* are repeated multiple times in tandem [10], allowing for efficient transcription by Pol I. Like in other eukaryotes, the current accepted mechanism of the production of the 18S, 5.8S and 25S components of the ribosome in this yeast, is transcription of a 35S copy of the rDNA, followed by post and co-transcriptional processing of the nascent RNA [11].

Processed RNA molecules will typically have a single phosphate on their 5′-end making them vulnerable to processive 5′ → 3′ exonucleases that digests only RNA that has a 5′-monophosphate end [12]. In fact, a major use of these enzymes is to help with mRNA purification by eliminating ribosomal RNA. We previously found resistant 18S and 25S rRNA molecules [13] after digesting *C. albicans* total RNA with such an exonuclease, Terminator (Lucigen)*.* Resistant ribosomal 18S and 25S behaved like 5S molecules, products of Pol III with triphosphates at their 5′ end, which we found to be resistant to Terminator digestion. However, 18S and 25S molecules were efficiently eliminated during mid-log growth phase but remained intact as cells approached the stationary growth phase. They differed from 5S, as 5S remains resistant to Terminator digestion throughout the growth cycle. Decapping, reaction that results in a single 5′ phosphate, reestablished these 18S and 25S molecules’ vulnerability to 5′-exonuclease digestion. This indicated that they contained more than a single phosphate at their 5′-end, which raised the possibility that they were newly transcribed. When we digested these Terminator resistant molecules with alkaline phosphatase, they remained resistant to Terminator digestion. This raised two possibilities: either they were 5′- triphosphated products and the alkaline phosphatase eliminated all three phosphates, leaving a 5′ hydroxyl end resistant to exonuclease digestion, or they were modified such as being capped, with the cap preventing both alkaline phosphatase and exonuclease digestion. If these molecules were indeed triphosphated and capped, that would still leave two possible scenarios: either they were newly transcribed Pol II products or were Pol I transcribed and processed molecules that were capped in the cytoplasm. Recapping in cytoplasm is well established for decapped mRNAs in mammalian cells [14] but has also been found in the eukaryote Trypanosoma brucei [15] . While the processed Pol I 18S and 25S transcripts that have single 5′-phosphates are not candidates for the canonical capping reactions (carried out by RNA triphosphatase and guanylyl transferase) [16], a cytoplasmic capping complex with kinase capabilities, capable in converting 5′-monophosphates to a GpppN 5′- terminus has been described [17]. Capping of ribosomal RNA components to preserve them for ribosome building would be by itself a novel finding. However, here we present data that in sum total raise the possibility of involvement by Pol II in the production of these resistant 18S and 25S molecules in *C. albicans.*

## Results

### 5′ end analysis of terminator-resistant 18S and 25S

**Fig. 1:**
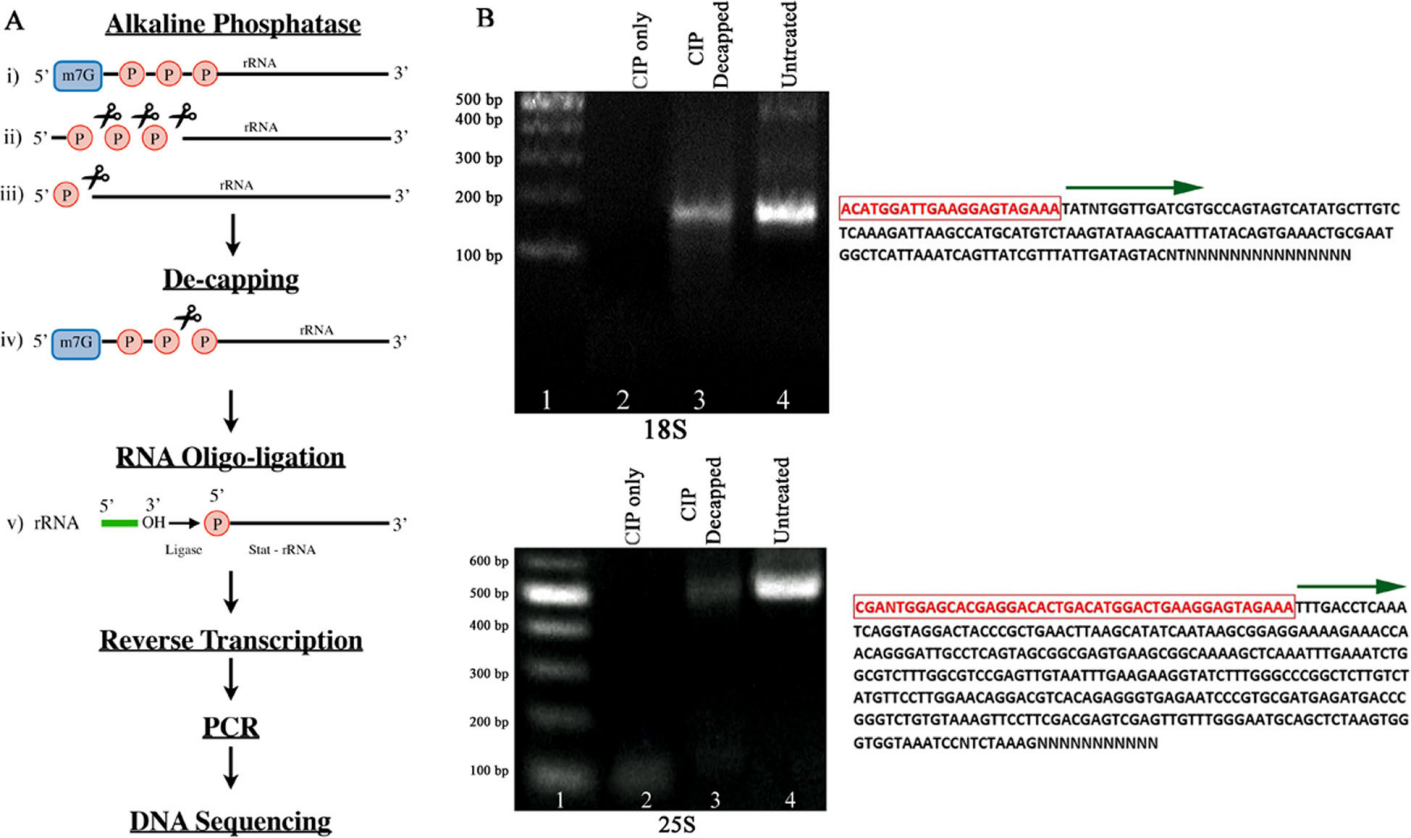
5′-end analysis of Terminator resistant 18S and 25S molecules. **a** Schematic representation of oligo ligation to indicate presence of more than one phosphate at the 5′-end of terminator resistant molecules. Ribosomal RNA is first treated with alkaline phosphatase, resulting in complete phosphate removal, if there is nothing protecting the triphosphate (ii and iii) or an intact cap-protected rRNA molecule (i). (iv) Use of the decapping enzyme CAP-Clip after CIP treatment makes a phosphate available for RNA oligo ligation (v) and indicates protection of the triphosphate molecules from CIP digestion. Reverse transcription using rDNA primers is performed followed by PCR and the amplicons of predicted size are sequenced. **b** SYBR-gold stained gel and sequences of PCR products amplified with oligo and internal specific primers (see Table S1) for 18S and 25S. Multiple amplifications and amplicons (at least 5) for each 18S and 25S generated same sequences. No amplifications were obtained for samples treated with CIP alone (lane 2). CIP followed by decapping (Lane 3) shows an amplification product of the predicted size. Untreated RNA (Lane 4) also resulted in the predicted amplicon size. Sequences of lane 3 products shown in detail. Red enclosed letters indicate oligo sequence. Green arrows indicate the 5′-end of these terminator resistant molecules being the same as the 5′-end of processed 18S and 25S [18]. RNA was extracted from stationary phase *C. albicans*

Ligation with T4 ligase, which requires a 5′ single phosphate on recipient molecule, was carried out by employing an RNA oligo with an OH group at the 3′-end. Having a 5′ single phosphate is also a requirement for the Terminator exonuclease to be able to digest RNA. Figure 1a illustrates how alkaline phosphatase digestion followed by a decapping reaction might affect the ligation possibilities. For both 18S and 25S molecules, multiple ligation attempts upon alkaline phosphatase digestion alone resulted in no products after primer extension and amplification. This indicated that any 5′ single phosphates, such as the ones on processed ribosomal molecules were eliminated, thus preventing oligo ligation. When alkaline phosphatase digestion was followed by decapping, PCR products of predicted sizes were obtained for both molecules (Fig. 1b). This again confirms that there was more than one phosphate at the 5′ end of these molecules. What was unexpected was to find upon sequencing, that the RNA oligo was attached at the reported processing sites of both molecules [18] (Fig. 1b).

### Quantification and cellular source of terminator-resistant 18S and 25S molecules

The finding of Terminator-resistant rRNA molecules by gel electrophoresis in cells during the stationary growth phase has remained consistent over many repeat digestions. These observations however, lacked in quantifying accurately the percentage of such molecules produced by the cells’ ribosomal RNA transcription system. To quantitate the amount of Terminator resistant 18S and 25S produced by the cells under different experimental conditions, such as mid-log and stationary growth phases, we used an Agilent 2100 Bioanalyzer. Additionally, as these Terminator resistant molecules clearly behaved differently than the usual 18S and 25S we wanted to see if they could be eliminated by Pol I inhibition. A small molecule termed BMH21 (N-[2-(Dimethylamino)ethyl]-12-oxo-2H-benzo [g]pyrido [2,1-b]quinazoline-4-carboxamide**)** identified through mammalian cell screens, was found to inhibit RNA polymerase I specifically by degrading its RPA194 subunit [19]. More recently, it was shown that this activity toward Pol I is conserved in yeast and conveniently, this molecule penetrates the cell wall, avoiding the need for spheroplasting [20]. To assess individual RNA components, total and nuclear RNA concentrations from mid-log, stationary cells and those exposed to the Pol I inhibitor were adjusted to equal amounts prior to Terminator digestion.

**Fig. 2:**
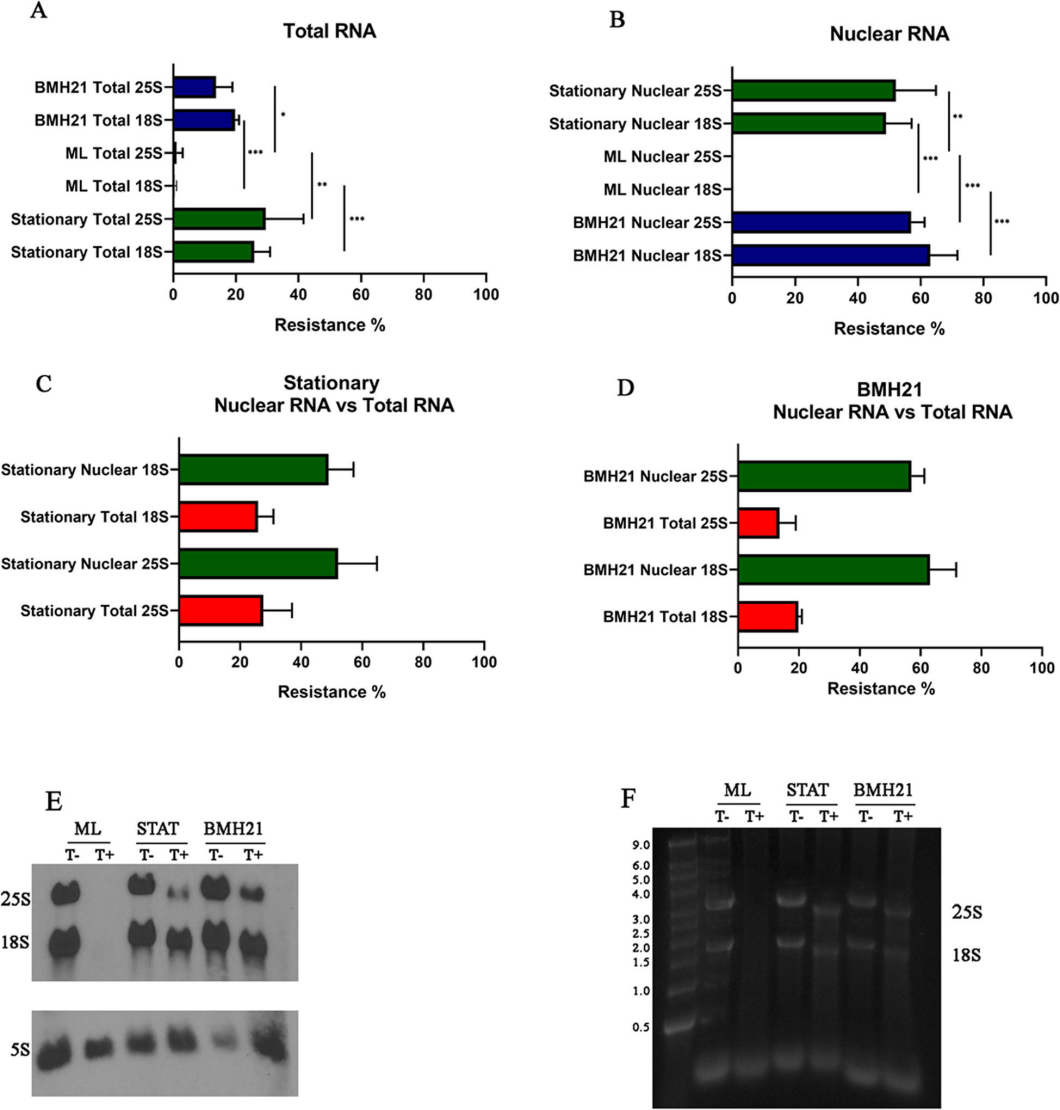
Percentage of Terminator resistant ribosomal RNA 18S and 25S molecules, as measured by Agilent Bioanalyzer 2100. **a** Resistance to Terminator digestion in total RNA isolated from of *C. albicans,* either from mid-log (ML) and stationary growth phases and mid-log cells treated with BMH21. **b** Terminator resistance percentage measured in RNA isolated from nuclei under same pre-isolation conditions as in (**a**). **c** Comparison of Terminator resistance percentages in nuclear versus total RNA from cells in stationary growth phase and (**d**) BMH21 treated cells. For each condition three different experiments were performed. Statistical analysis was done using Agilent Bioanalyzer 2100 Expert Software. *P* values generated by Student’s test, *p < 0.05, **p < 0.01, ***p < 0.001. **e** Northern blot showing RNA extracted from *C. albicans* at mid-log (ML), stationary (STAT) and after BMH21 treatment. RNA was digested with Terminator (T+) or undigested (T-) and the membrane was hybridized with 25S, 18S and 5S probes (Table S1) **f** SYBR gold stained agarose gel that was used in Northern blot in (**e**)

As seen in Fig. 2a, total RNA extracted from both cells in stationary phase and those inhibited by BMH21 contained significantly more amounts of Terminator resistant 18S and 25S when compared to cells in mid-log growth phase. This was also true in RNA obtained from the nuclei (Fig. 2b). Comparisons of nuclear versus total RNA (Fig. 2c and d) showed that predominantly, these molecules are produced in the nuclei both in stationary and BMH21 inhibited cells. This makes it likely that majority of the resistant 18S and 25S molecules were synthesized in the nucleus. Furthermore, the fact that they appeared in the nucleus upon Pol I inhibition argues for another system responsible for their synthesis. Analysis for histone acetyltransferase activity (HAT) showed that the source of the nuclear RNA was in fact the nucleus (Fig. S4). Also, the fact that we see more Terminator resistance in the nuclear RNA makes it unlikely that this is due to cytoplasmic contamination. Terminator resistance can result from additional phosphates at the 5′-end of molecules as shown by 5S resistance to Terminator digestion (Fig. 2e and f). Therefore, the complete digestion by Terminator of 18S and 25S from mid-log phase organisms indicates that they consist primarily of single phosphates at their 5′-end. In contrast, both stationary and BMH21 treated cells contain 18S and 25S Terminator resistant, similar to 5S.

### Presence of caps on 18S and 25S in RNAs from stationery and pol I inhibited cells

To see if there are any Terminator resistant molecules that have a 7-methylguanosine cap, and thus likely to be a Pol II product, we used monoclonal antibodies that were generated by a carrier protein-conjugated 7-methylguanosine (m^7^G)-cap analogue (designated as M7AB or H20). These antibodies have the most avidity for the 5′-terminal cap but do partially cross react with m^7^G within RNA.

**Fig. 3:**
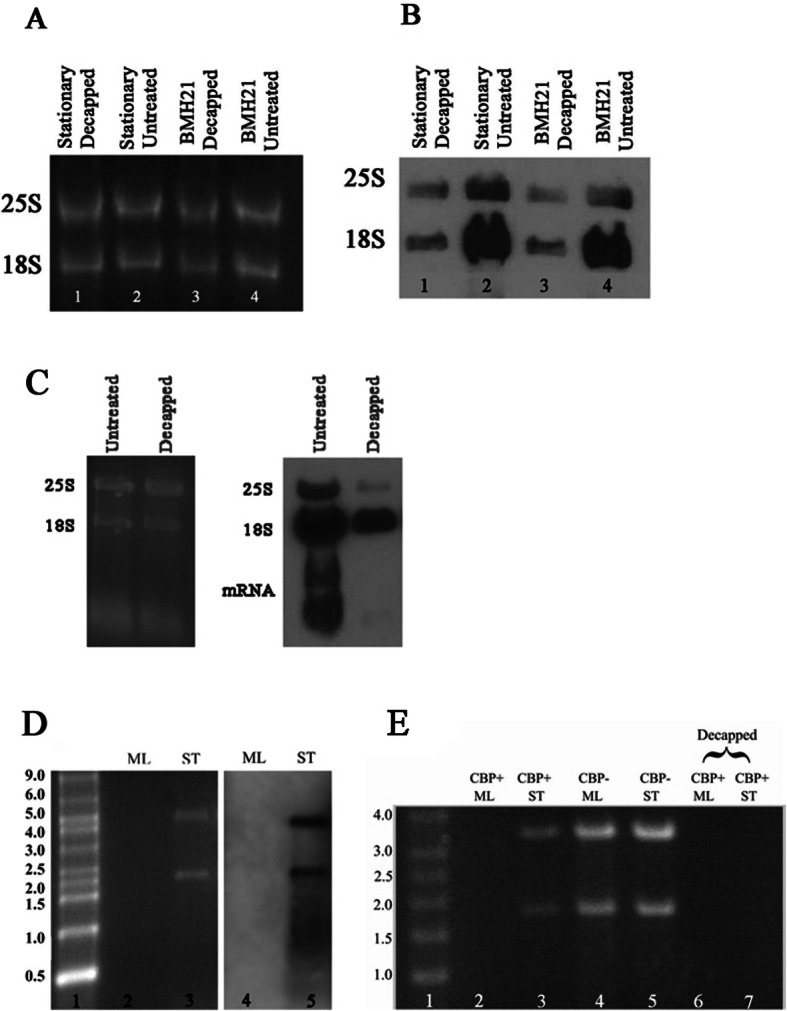
Presence of caps on 18S and 25S RNAs from stationary and BMH21 treated cells. **a** SYBR-gold stained gel showing that decapping did not affect RNA integrity. **b** Immunoblot of the gel (**a**) showing decrease in intensity after treatment with decapping enzyme. Some cross reactivity can be seen in lanes 1 and 3 (see text). **c** SYBR gold stained gel and corresponding Northern blot showing decapping of 18S, 25S and mRNAs. **d** SYBR gold stained gel showing total RNA extracted from mid-log (ML) and stationary (ST) *C. albicans* (lanes 2 and 3) and immunoblot (lanes 4 and 5) using anti-m7G-cap mAb to detect bands precipitated by cap binding protein (CBP) eIF4F. **e** Conditions in lanes 2 and 3 are the same as in (**d**), lanes 4 and 5 are untreated mid-log and stationary RNA. In lanes 6 and 7 RNA was decapped prior to precipitating with CBP, showing that decapping removes the target of eIF4E protein

Evidence for a 5′ cap can be seen in Fig. 3a and b where Terminator-resistant rRNAs from stationary and BMH21 treated cells were subjected to decapping reaction. As can be seen on the SYBR-Gold stained gel (Fig. 3a), decapping did not change the total RNA content. Yet there is a significant decrease in the intensity of the signal generated by the antibody after decapping for both conditions and both 18S and 25S (Fig. 3b). Figure 3c shows that decapping enzyme works efficiently on capped mRNAs.

Another approach to show a 5’cap was to combine the anti-cap antibody with a cap-binding protein. His-tagged eIF4E, a subunit of eIF4F cap binding protein complex (CBP) was used to see if it would precipitate any 18S and 25S molecules. eIF4F has special affinity for 5’cap and is unlikely to be cross reacting with parts of an RNA molecule other than the 5’cap. RNA from stationary cells indeed contained such molecules and none could be seen in RNA from mid-log organisms (Fig. 3d, lanes 2 and 4). Anti-cap antibody reacted to these molecules (Fig. 3d, lane 5), further confirming that they contained 5’cap. Removing the cap by a decapping reaction prevented their precipitation (Fig. 3e, lanes 6 and 7), by eIF4F.

**Fig. 4:**
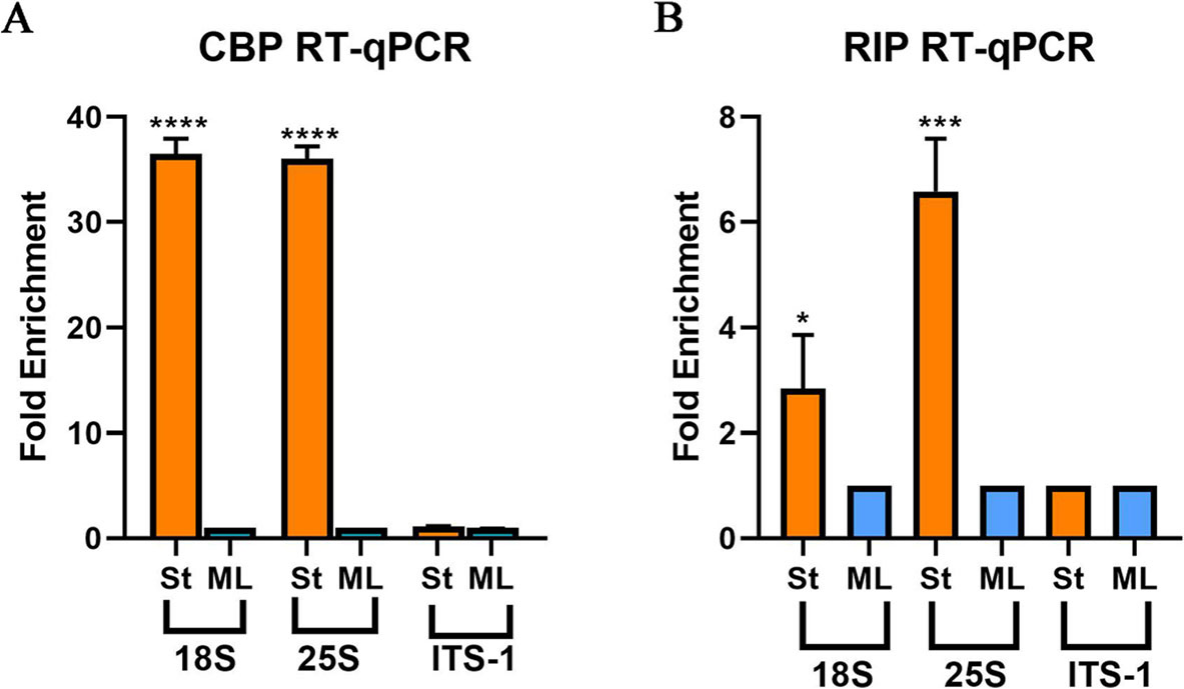
CBP and RIP qPCR analysis. (**a** and **b**) RT-qPCR quantification of 18S, 25S and ITS-1 molecules precipitated from total RNA by (**a**) eIF4E (CBP) and (B) anti-m7G-cap mAb, both from mid-log (ML) and stationary (St) organisms. ITS-1 was used as a negative control for the assay. Error bars represent standard deviation from three different experiments. *P* values generated by Student’s test, *p < 0.05, **p < 0.01, ***p < 0.001, ****p < 0.0001

The eIF4E precipitated RNA was also subjected to qPCR amplification with 18S and 25S specific primers. As controls we used primers for ITS1, clearly a product of Pol I and processing. The results can be seen in Fig. 4a. For both 18S and 25S significant amplification occurred only in RNA isolated from stationary stage. RNA immunoprecipitation (RIP) was also performed on the RNA utilizing M7AB antibody followed by qPCR, with the same ITS1 control and the results are shown on Fig. 4b. There was some amplification in RNAs from mid-log growth phase and ITS1 as would be expected as the antibody does cross react with m^7^G within the RNAs, but clearly there was a significant fold enrichment on RNA from stationary organisms. These PCR amplifications also point toward the presence of a cap in the 5′ position in stationary cells since RT-qPCR was performed on RNA molecules precipitated by either a cap specific antibody or a cap binding protein.

### RT-qPCR measurements of 18S and 25S transcription rates on pol I inhibition

**Fig. 5:**
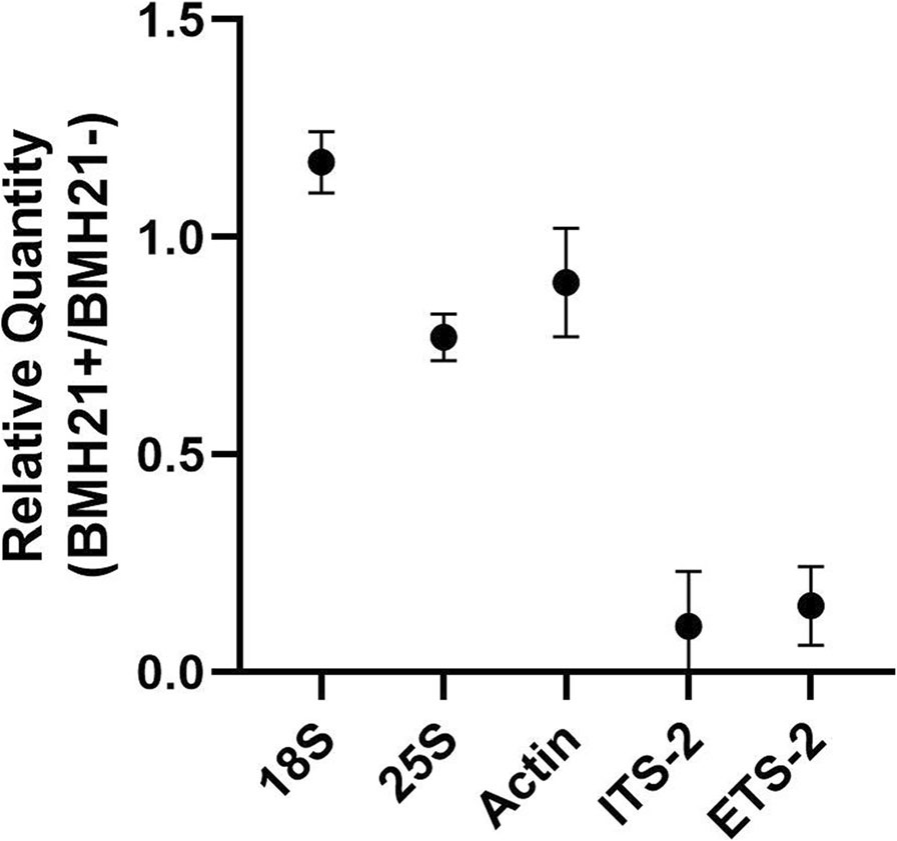
RT-qPCR measurement of 18S and 25S transcription rate on Pol I inhibition. Relative quantity was obtained by calculating the ratio of the number of amplicons produced in *C. albicans* with and without BMH21 exposure. Bars represent standard deviations based on four different experiments

Figure 5 shows the combined results of multiple qPCR amplifications utilizing Pol I specific primers for 5′-ETS and ITS2 and comparing them to primers specific for 18S and 25S. As can be seen, the number of amplicons produced on BMH21 inhibition, is significantly diminished for 5′-ETS and ITS2, not so for 18S and 25S. Ratios for Actin indicate that Pol II was not inhibited by BMH21. If 18S and 25S were exclusive products of Pol I transcription, ETS and ITS2 ratios should mirror those of 18S and 25S. These results indicate that BMH21 does inhibit Pol I in *C. albicans* and some of the 18S and 25S molecules are transcribed by something other than Pol I.

### Production of terminator resistant 18S and 25S upon TOR inhibition

**Fig. 6:**
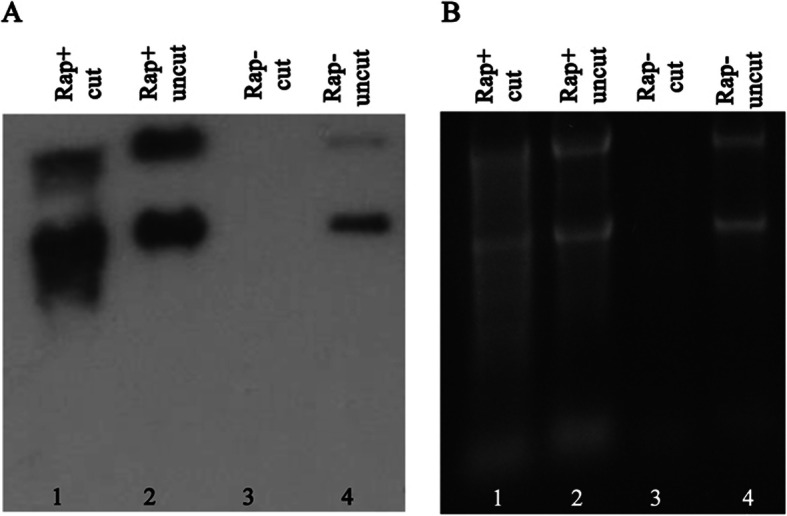
Rapamycin induces Terminator resistance in RNA molecules. **a** Immunoblot of gel shown in (**b**) was done using M7AB antibody. **b** SYBR-Gold stained gel of RNA extracted from *C. albicans* incubated with (Rap+) (lanes 1 and 2) and without rapamycin (Rap-) (lanes 3 and 4) for 5 h was digested (lanes 1 and 3) or not digested (uncut) (lanes 2 and 4) with Terminator exonuclease

As we have previously indicated, rapamycin inhibition leads to cells producing Terminator-resistant 18S and 25S [13]. Using immunoblotting analysis, we now show that 18S and 25S produced after TOR inhibition can be detected by an anti-cap antibody (Fig. 6). Figure 6a represents an immunoblot analysis of these molecules with Fig. 6b showing a SYBR-Gold stained gel from which the immunoblot was obtained. Figure 6a, lanes 2 and 4, while indicating some expected cross reactivity with the untreated molecules, show a multifold increase in intensity of the bands representing RNA from rapamycin treated cells. Furthermore, Terminator treatment did not significantly reduce band intensities (Fig. 6a lane 1) of treated cells, while eliminating RNA from untreated cells (Fig. 6a lane 3). Figure 6b indicates that the intensity differences seen on the immunoblot (lanes 2 and 4) were not the result of different amounts of RNA loaded.

### Chromatin immunoprecipitation analysis to detect presence of pol II on rDNA

**Fig. 7:**
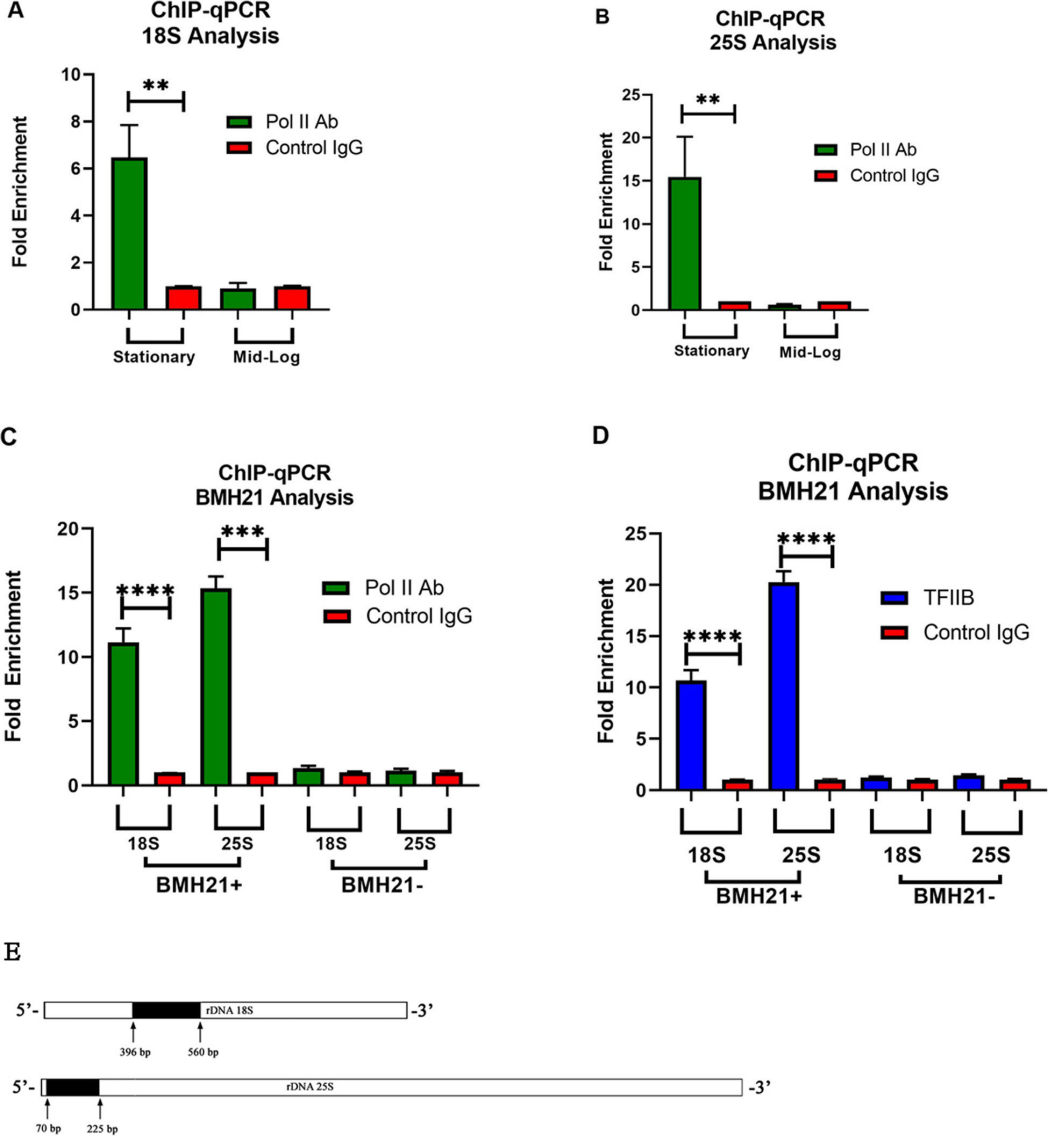
Chromatin immunoprecipitation (ChIP) analysis. ChIP places Pol II on rDNA 18S and 25S sequences during stationary growth phase and on BMH21 stimulation. (**a-d**) Chromatin immunoprecipitation with polymerase II and TFIIB specific antibodies. Cells were cross-linked, and chromatin was sheared by sonification. RNA Polymerase II mAb CTD4H8 (Epigentek) was used to precipitate DNA-protein complex. qPCR was performed using two different sets of specific primers, 18S and 25S. A control (non-immune) IgG antibody was used as negative control. BMH21- indicates mid-log cells not exposed to BMH21. Error bars represent standard deviation from three different experiments. *P* values were generated by Student’s test, **p < 0.01, ***p < .0002, ****p < .0001. **e** Amplicons location on rDNA gene. See Supplementary Table 1 for primer information

The inhibitor BMH21 pointed to Pol I not being the source producing the Terminator-resistant molecules. The possibility of caps that are present on these molecules suggested Pol II as the source for these products. Therefore, we wanted to see if we could detect an increased presence of Pol II on the 18S and 25S regions of the ribosomal genes during periods when Terminator-resistant molecules are produced. To this end we performed chromatin precipitation with monoclonal antibody CTD4H8 widely used for Pol II recognition [21]. Results of qPCR amplifications with 18S and 25S specific primers can be seen in Fig. 7. DNA precipitated with the specific antibody resulted in a significant fold enrichment over the non-specific antibody only in DNA isolated from cells in the stationary phase and not in those from mid-log phase (Fig. 7a and b). This was true for both primers specific for 18S and 25S. Similarly, no bands could be generated from DNA extracted with non-specific antibody by routine PCR (Fig. S5, lanes 4–6 and 11–13). Results for DNA isolated from mid-log cells exposed to BMH21 (Fig. 7c) show a similar fold increase when specific and non-specific antibodies are compared, but not seen in mid-log cells not exposed to BMH21. Mid-log cells were chosen for the obvious reason that unlike stationary cells they don’t produce Terminator-resistant molecules on their own. These data indicate the presence of Pol II on 18S and 25S rDNA at the same period when Terminator-resistant molecules are detected. In addition to Pol II specific antibody, we also performed ChIP analysis with antibody specific to TFIIB initiation factor. The results can be seen in Fig. 7d where a significant fold enrichment is observed for both 18S and 25S after TFIIB precipitation.

## Discussion

Our data establish that *C. albicans* can produce two different populations of 18S and 25S components of ribosomal RNA. One group is produced by the currently accepted mechanism of polycistronic transcription of the rDNA by Pol I, followed by processing into the components. These molecules are highly susceptible to digestion by the 5′ phosphate requiring exonuclease Terminator. The second group mimics the behavior of the 5S molecule in resisting Terminator digestion. The 5S molecule is newly transcribed by Pol III with a resultant triphosphate at its 5′ end [22] and it is Terminator-resistant [13]. The fact that 18S and 25S rRNAs became Terminator susceptible upon decapping, shows the presence of additional phosphate(s) at their 5′-end. Like Terminator, ligation of an RNA oligo requires a single 5′ phosphate on recipient RNA and occurred on alkaline phosphatase treated RNA only after decapping of 18S and 25S (Fig. 1b). This again confirmed the extra phosphates at the 5′-end. Our data indicating that the majority of these Terminator resistant molecules, with more than a single phosphate at their 5′-end, originate from the nucleus, argues against them being modified processed molecules, as such modifying systems are located in the cytoplasm [14]. The higher percentage of Terminator resistant rRNA molecules from material isolated from the nucleus assures us that they do not represent cytoplasmic contamination. Thus, having more than a single 5′-phosphate on these 18S and 25S molecules emerging from the nucleus favors new transcription. Quantitative PCR measurements of 18S and 25S during conditions favoring Terminator resistance (Fig. 5) also point to new transcriptions of some of these molecules.

What we know from many studies of Pol I and its promoter complex [3], makes it unlikely that Pol I would initiate transcription of these molecules. The fact that inhibition of Pol I with BMH21 induced the production of Terminator resistant molecules in the nucleus also suggests that Pol I is not their transcriber. Several approaches converge to raise the possibility of a role for Pol II in the synthesis of resistant 18S and 25S molecules. This includes chromatin immunoprecipitation, and the possible presence of caps on Terminator resistant molecules, as shown by cap specific antibody and cap binding protein, both by visual (Fig. 3) and amplification methods (Fig. 4).

While ribosome generation has not been a focus in *C. albicans*, studies in *Saccharomyces cerevisiae* and other yeast has been extensive and should be relevant to our organism. As already mentioned, Pol II is capable of transcribing rRNA, which has been shown in *S. cerevisiae* [7]. When *RRN9*, one component of the Pol I upstream activating factor (UAF) was deleted, inactivating Pol I, the yeast was capable of ribosome production utilizing Pol II. Transcription was initiated from multiple start sites upstream or downstream from the normal Pol I’s promoter site, still in a polycistronic fashion. It has also been seen in a petite strain of *S. cerevisiae,* involving the selective activation of cryptic Pol II promoters from episomal rDNA elements [8]. The rDNA tandem array, concentrated in nucleoli of yeast where Pol I is active, is a gene silencing region for Pol II activity [23]. It differs from mating loci and telomere silencing regions, in that active suppression of Pol II coexists with highly active transcription by Pol I. While several mechanisms have been proposed for this paradoxical observation, multiple observations, combined with reporter *mURA3* gene integration studies have led to a model of “reciprocal silencing” [24]. That is, chromatin conditions favoring Pol I, decrease or silence Pol II and vice versa. The Pol I transcribed rDNA repeats are separated by non-transcribed sequences (NTS) divided by the 5S rRNA gene. Molecular studies have localized rRNA transcription silencing of Pol II to these interweaving sequences. This is where NAD^+^-dependent histone deacetylase Sir2, as part of the RENT complex is attracted and concentrated, leading to repressive chromatin structure changes [25]. However, still Pol II can gain access even to the non-transcribed sequences as indicated by its ability to copy non-coding RNAs [26]. Should our data be confirmed it would point to another possible example of Pol II escaping silencing and participating in rRNA production for the cell. It would indicate that Pol II can get access by a hitherto unknown mechanism to rDNA, downstream from the Sir2 silenced NTS and guided specifically to or near the processing site. The previously mentioned study [7] where *RRN9*, one component of the Pol I upstream activating factor (UAF) was deleted allowing Pol II to initiate transcription from multiple sites, indicates incidentally that Pol II does not always require a functional promoter to initiate transcription.

Target of rapamycin (TOR) signal transduction pathway regulates ribosome production including the transcription and processing of 35S rRNA [27]. As nutritional sources of the cell ebb or when expose to rapamycin, changes in TOR activity decrease Pol I activity, eventually displacing it from the nucleolus [28]. This could allow Pol II access to rDNA repeats in the nucleolus. Our data of Terminator resistance developing upon rapamycin inhibition of TOR (Fig. 6) is compatible with this possibility.

The Terminator assay establishes that *C. albicans* can produce two different populations of 18S and 25S ribosomal RNA components. We also show evidence from multiple directions that Pol II may be involved in this process though more direct evidence will be needed to firmly establish this role for this enzyme complex. This is especially true as our oligo-ligation and primer extension mapped the 5′-end of these resistant molecules at or near the processing site and we fully appreciate that to accept that Pol II should start transcribing there will need additional proof. Should this role be confirmed, it would add two more molecules to the others known to be transcribed by Pol II.

Whether capped or newly transcribed, the production of these 18S and 25S molecules appears to function as a backup system for the cell during unfavorable nutritional states to maintain some capacity for protein production. Our previous finding that such molecules were incorporated into ribosomes [13] further supports this idea. Indeed, *C. albicans* expresses genes specifically in the stationary phase that play important roles in pathogenesis [29]. It is of interest that we have found similar exonuclease resistant 18S and 25S molecules in *S. cerevisiae* and the fission yeast *Schizosaccharomyces pombe* [13]. It is unknown whether such a system is present beyond those of yeasts. Clearly, an additional system maintaining the production of ribosomes, and therefore proteins, would be advantageous for cells with high metabolic requirements such as malignant cells [30].

## Conclusions

We feel confident that in this yeast there can be two types of 18S and 25S ribosomal RNA components as they have been consistently found on hundreds of Terminator digestions. Decapping these resistant molecules re-establishes their susceptibility to Terminator indicating that an extra phosphate is part of their 5′-end modification. The data we present now hint at the unexpected finding that these changes may be as a result of new transcription and equally surprising by Pol II and pursuing the validity of this possibility is worthwhile. Should this be definitively proven, it would establish another role for RNA Polymerase II in the production of ribosomes. Just as interesting would be to elucidate the nature of the promoter allowing Pol II to carry out these transcriptions.

## Methods

### Organisms

*Candida albicans* SC5314 (purchased from ATCC MYA 2876) was maintained in 50% glycerol in YPD broth (2% w/v tryptone, 1% w/v yeast extract, 2% w/v dextrose) at − 80 °C. Cells were activated in YPD broth at 30 °C and maintained on Sabouraud dextrose agar at 4 °C, passaged every 4–6 weeks up to 4–5 times. Yeasts were lifted from agar surface and grown in YPD broth for variable length of times at 30 °C. Yeast cell concentrations were established using a hemocytometer.

### RNA isolation

Cells were collected by centrifugation, washed with sterile phosphate buffered saline (PBS) and were put on ice pending total RNA extraction. Cells were disrupted with RNase-free zirconia beads and RNA was isolated using Ambion RiboPure RNA Purification kit for yeast (Ambion/ThermoFisher) according to the manufacturer’s instructions.

Nuclear RNA was obtained using the Yeast Nuclei Isolation kit (Abcam) following the manufacturer’s instructions. The quality of the nuclei was checked by adding DAPI (1:1) to 4 μg of the nuclear extract and observed under a fluorescent microscope. RNA quantification and quality were assessed by using a Qubit 2.0 fluorometer and an Agilent 2100 Bioanalyzer.

### RNA analysis

Terminator treated and non-treated RNA samples were loaded into an RNA 6000 Nano chip and analyzed with the Agilent 2100 bioanalyzer system (Agilent Technologies, INC). Terminator resistance percentages were calculated by measuring the areas of peaks representing ribosomal RNA components on electropherograms (Fig. S1-S3).

### Immunoblotting

RNA was separated on prefabricated formaldehyde agarose gels (Lonza) and stained with SYBR Gold Nucleic Acid Gel Stain (Life Technologies) for 30 min. Gel images were captured with a digital camera (Canon Vixia HFS30). RNA was transferred by electro-blotting (Thermo Scientific Owl Hep-1) to a positively charged nylon membrane (Life Technologies) in 0.5 x TBE (standard Tris/Borate/EDTA buffer). The RNA was cross-linked to the membrane using UV Crosslinker (Stratagene). Membrane was blocked with 10% Block Ace™ (Bio-Rad) for 30 min at 25 °C, followed by the addition of cap-specific monoclonal antibodies, either M7AB (MBL) or H20 (Millipore Sigma) diluted 1:1000 in 10% Block ACE™ and incubated for 24 h at 4 °C. Goat anti-mouse conjugated to HRP was added to the membrane at 1:5000 in blocking solution for 30 min at 25 °C. The Supersignal™ West Femto (Thermo Scientific) chemiluminescence substrate was used to detect the HRP signal. Film was developed with the SRX-101A Konica film processor.

### Terminator 5′-phosphate-dependent exonuclease experiments and 5′-end analysis

Total RNA was treated with Terminator (Lucigen) following the manufacturer’s protocol using the supplied Buffer A. The ratio of enzyme to substrate employed was 1 U per 1 μg of RNA to ensure adequate cleavage. Analysis of 5′-end rRNA was done by first digesting total RNA with alkaline phosphatase (New England Biolabs), followed by CAP-Clip Acid Pyrophosphatase (Cellscript) treatment. Resulting RNA was ligated to a GeneRacer™ oligo (see Table for sequence) using T4 RNA ligase followed by reverse transcription using rRNA primers. PCR was carried out with previously obtained cDNA as template using a DNA sequence similar to the ligated RNA oligo as forward primer, and for reverse primer a complement of the rDNA. Amplified PCR fragments of predicted size were sent out for sequencing using primers specific to 18S and 25S.

### Cap binding protein assay

Two micrograms of total RNA from *C. albicans* were incubated with 1 μg of recombinant human eIF4E protein fused to His-tag at N-terminus (Creative BioMart) in binding buffer (25 mM Tris, pH 8.0, 150 mM NaCl, 1 mM DTT, 5 mM imidazole) and incubated at 4 °C overnight. HisPur™ Ni-NTA magnetic beads (ThermoFisher Scientific) were added to the RNA-eIF4E mixture and placed on ice for 10 min. A magnetic stand was used to collect the beads after three washes with binding buffer. Capped RNA was eluted with 200 mM imidazole buffer. Finally, a phenol chloroform extraction was done in order to remove eIF4E protein off the eluate.

### Inhibitor assays

RNA polymerase I inhibitor *N*-[2-(Dimethylamino)ethyl]-12-oxo-12*H*-benzo [*g*]pyrido [2,1-*b*]quinazoline-4-carboxamide (BMH21) (Tocris), shown to be inhibiting for yeast was used at a 50 μM concentration. mTOR inhibitor, Rapamycin (Sigma) was used at the ratio of 1 μg per 1 × 10^6^ cells. Incubation time for both inhibitors was 60 min at 30 °C with constant shaking. After incubation cells were washed with PBS and used for the appropriate assay.

### RT-qPCR

RT-qPCR was carried out using 18S and 25S specific primers. Actin primers were used as negative controls for pol I whereas ITS-2 and 5′-ETS were used as positive controls (Table S1). RT-qPCR was done utilizing the qPCRBIO SyGreen 1-step Detect Lo-ROX (Genesee Scientific) kit following the manufacturer’s instructions.

### Decapping assays

Cap-Clip™ acid pyrophosphatase (Cellscript) was used according to manufacturer instructions for decapping RNA samples. Verification of cap removal was done by gel electrophoresis, immunoblotting and immuno-precipitation with cap binding protein.

### RNA immunoprecipitation and amplification

RNA immunoprecipitation (RIP) was performed on mid-log and stationary RNA with anti-m7G-Cap mAb and protein A/G magnetic beads to purify the antibody-capped RNA complex. RNA was extracted with Direct-zol™ (Zymo Research). RT-qPCR was carried out using 18S and 25S specific primers. Positive and negative controls were actin and ITS-1 respectively (Table S1).

### RNA precipitation and amplification using cap binding protein

RNA precipitation was performed on mid-log and stationary RNA with his-tagged cap binding eIF4e protein and HisPur™ Ni-NTA magnetic beads as described in the CBP section. RT-qPCR was done using 18S and 25S specific primers. Positive and negative controls were actin and ITS-1 respectively (Table S1).

### Native chromatin immunoprecipitation (ChIP) analysis

*C. albicans* (1 × 10^6^ c/mL) were grown at 30 °C for 16 h (stationary) in a 500 mL YPD. Crosslinking was done by adding formaldehyde to the culture and incubated at RT for 20 min with gently swirling. After that, 37.5 mL of 3 M glycine, 20 mM Tris was added and incubated for 5 min. Cells were pelleted at 2000 rpm for 5 min and washed twice with 200 mL cold TBS (20 mM Tris-HCl, pH 7.5, 150 mM NaCl) and once with 10 mL cold FA lysis buffer (100 mM Hepes-KOH, pH 7.5, 300 mM NaCl, 2 mM EDTA, 2% Triton X-100, 0.2% Na Deoxycholate)/0.1% SDS. Pellets were resuspended in 1 mL cold FA lysis buffer/ 0.5% SDS. Cells were broken up by Zirconia bead (Ambion) vortexing. Chromatin isolation and shearing were done following Keogh and Buratowski [31]. Isolation of protein/DNA fragments specific for RNA polymerase II were selected with the ChromaFlash High Sensitivity ChIP Kit (Epigentek) with antibody specific for RNA polymerase II c-terminal domain, following the manufacturer’s instructions. TFIIB antibody (Santa Cruz Biotechnology) was used at a dilution of 1:1000. PCR analysis was done to confirm the presence of protein/DNA complexes containing 18S and 25S RNA specific sequences. PCR amplicons were sequenced (Laragen Inc.) using the same reverse primers as the ones utilized for the PCR (see Table S1).

### Quantification and statistical analysis

Statistical analysis was done using GraphPad Prism software (version 8.2). *p* values were calculated using two tailed unpaired t test and were considered statistically significant when they were less than 0.05.

## Supplementary information

**Additional file 1 Fig. S1**. Representative electropherograms used to calculate Terminator resistance percentages in 18S and 25S. The areas under each peak were obtained using the Bioanalyzer Expert software. These areas were used to calculate the percentage of RNA resistance by obtaining the ratio between cut (Terminator treated) and uncut (untreated) RNA. **a** total RNA from mid-log organisms untreated and **b** treated with Terminator. **c** nuclear RNA from mid-log organisms untreated and **d** treated with Terminator. **Fig. S2**. Representative electropherograms used to calculate Terminator resistance percentages in 18S and 25S. The areas under each peak were obtained using the Bioanalyzer Expert software. These areas were used to calculate the percentage of RNA resistance by obtaining the ratio between cut (Terminator treated) and uncut (untreated) RNA under different conditions. **a** total RNA from stationary organisms untreated and **b** treated with Terminator. **c** nuclear RNA from stationary organisms untreated and **d** treated with Terminator. **Fig. S3**. Representative electropherograms used to calculate Terminator resistance percentages in 18S and 25S. The areas under each peak were obtained using the Bioanalyzer Expert software. These areas were used to calculate the percentage of RNA resistance by obtaining the ratio between cut (Terminator treated) and uncut (untreated) RNA under different conditions. **a** total RNA from BMH21 exposed organisms untreated and **b** treated with Terminator. **c** nuclear RNA from BMH21 exposed organisms untreated and **d** treated with Terminator. **Fig. S4.** Histone Acetyltransferase Activity Assay (HAT) results. Nuclear extracts from *C. albicans* were compared at three different concentrations to a positive extract (control). HAT assays were carried out in order to verify that the nuclear RNA source was indeed the nucleus. **Fig. S5.** Evidence for the role of RNA Pol II in the transcription of 18S and 25S molecules in stationary *C. albicans.* Chromatin Immunoprecipitation (ChIP) with polymerase II specific antibody. PCR fragments amplified from stationary organisms. Cells were cross-linked and chromatin was sheared by sonification. RNA Polymerase II mAb CTD4H8 (Epigentek) was used to precipitate DNA-protein complex. PCR was performed using three different sets of specific primers for 18S (PO-PB, PA-PP, PK-PQ) and 25S (PR-PD, PC-PS, PL-PT) (+). See Supporting Table 1 for primers information. A non-immune IgG antibody was used as negative control (−). **Table S1.** List of primers used in all the experiments

## Data Availability

The datasets generated and/or analyzed during the current study are available in the DRYAD repository 10.5068/D16H37

## Abbreviations

Pol I: RNA Polymerase I
Pol II: RNA Polymerase II
Pol III: RNA Polymerase III
UAF: Upstream activating factor
CIP: Calf Intestinal Phosphatase
BMH21: (N-[2-(Dimethylamino)ethyl]-12-oxo-2H-benzo [g]pyrido [2,1-b]quinazoline-4-carboxamide)
HAT: Histone acetyltransferase
ML: Mid-log
STAT: Stationary
CBP: Cap binding protein
RIP: RNA immunoprecipitation
ChIP: Chromatin immunoprecipitation
TOR: Target of rapamycin
TFIIB: Transcription factor IIB
NTS: Non-transcribed sequences;
