# Supplementary material for: Exonuclease resistant 18S and 25S ribosomal RNA components in yeast are possibly newly transcribed by RNA polymerase II
Source: BMC Mol Cell Biol. 2020 Aug 1;21:59. doi: 10.1186/s12860-020-00303-z (PMC7395337; doi:10.1186/s12860-020-00303-z)
Supplement: Supplementary file 1 — Additional file 1 Fig. S1. Representative electropherograms used to calculate Terminator resistance percentages in 18S and 25S. The areas under each peak were obtained using the Bioanalyzer Expert software. These areas were used to calculate the percentage of RNA resistance by obtaining the ratio between cut (Terminator treated) and uncut (untreated) RNA. a total RNA from mid-log organisms untreated and b treated with Terminator. c nuclear RNA from mid-log organisms untreated and d treated with Terminator. Fig. S2. Representative electropherograms used to calculate Terminator resistance percentages in 18S and 25S. The areas under each peak were obtained using the Bioanalyzer Expert software. These areas were used to calculate the percentage of RNA resistance by obtaining the ratio between cut (Terminator treated) and uncut (untreated) RNA under different conditions. a total RNA from stationary organisms untreated and b treated with Terminator. c nuclear RNA from stationary organisms untreated and d treated with Terminator. Fig. S3. Representative electropherograms used to calculate Terminator resistance percentages in 18S and 25S. The areas under each peak were obtained using the Bioanalyzer Expert software. These areas were used to calculate the percentage of RNA resistance by obtaining the ratio between cut (Terminator treated) and uncut (untreated) RNA under different conditions. a total RNA from BMH21 exposed organisms untreated and b treated with Terminator. c nuclear RNA from BMH21 exposed organisms untreated and d treated with Terminator. Fig. S4. Histone Acetyltransferase Activity Assay (HAT) results. Nuclear extracts from C. albicans were compared at three different concentrations to a positive extract (control). HAT assays were carried out in order to verify that the nuclear RNA source was indeed the nucleus. Fig. S5. Evidence for the role of RNA Pol II in the transcription of 18S and 25S molecules in stationary C. albicans. Chromatin Immunoprecipitation ( [file 12860_2020_303_MOESM1_ESM.docx]

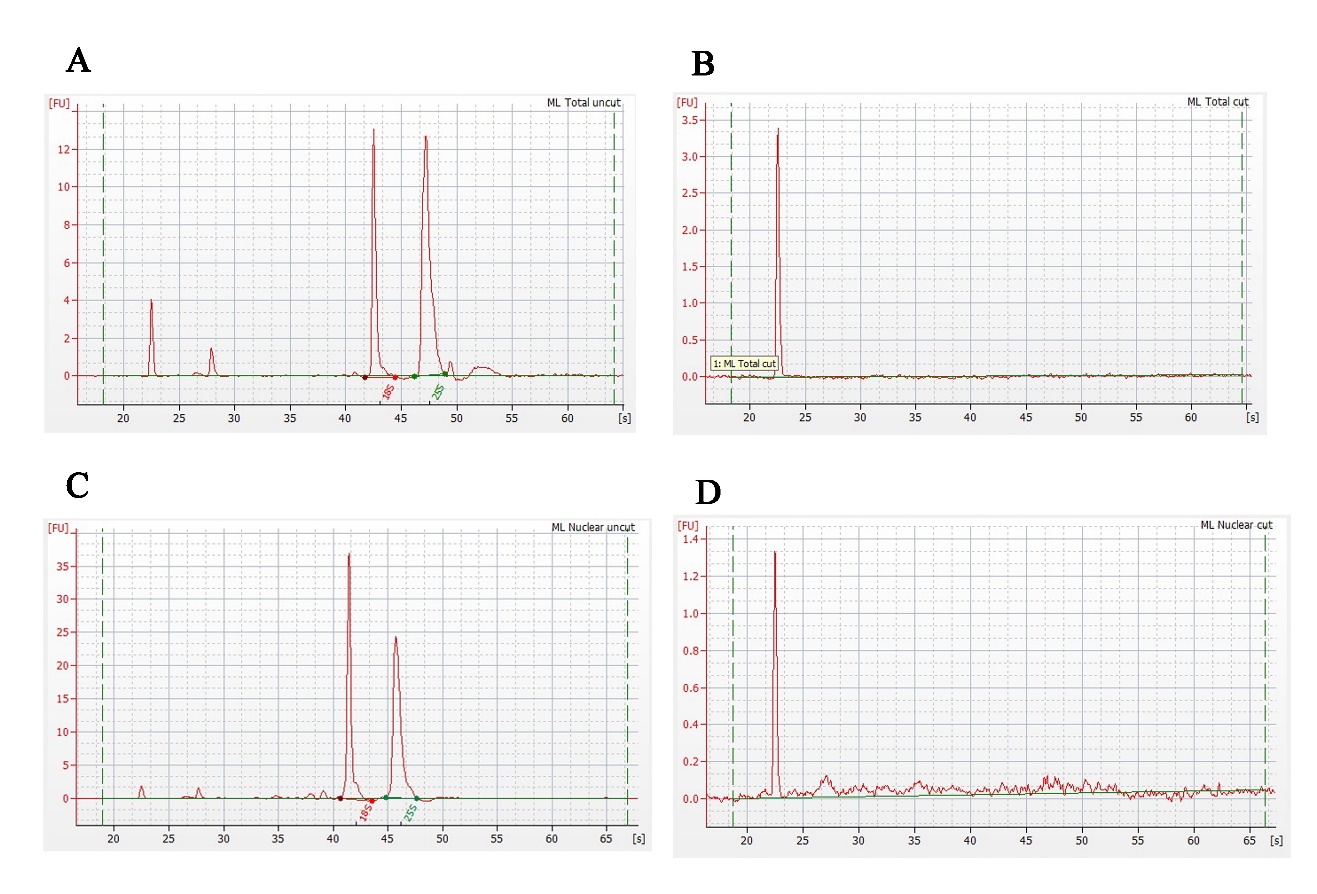


**Fig S1**. Representative electropherograms used to calculate Terminator resistance percentages in 18S and 25S. The areas under each peak were obtained using the Bioanalyzer Expert software. These areas were used to calculate the percentage of RNA resistance by obtaining the ratio between cut (Terminator treated) and uncut (untreated) RNA. **a** total RNA from mid-log organisms untreated and **b** treated with Terminator. **c** nuclear RNA from mid-log organisms untreated and **d** treated with Terminator.


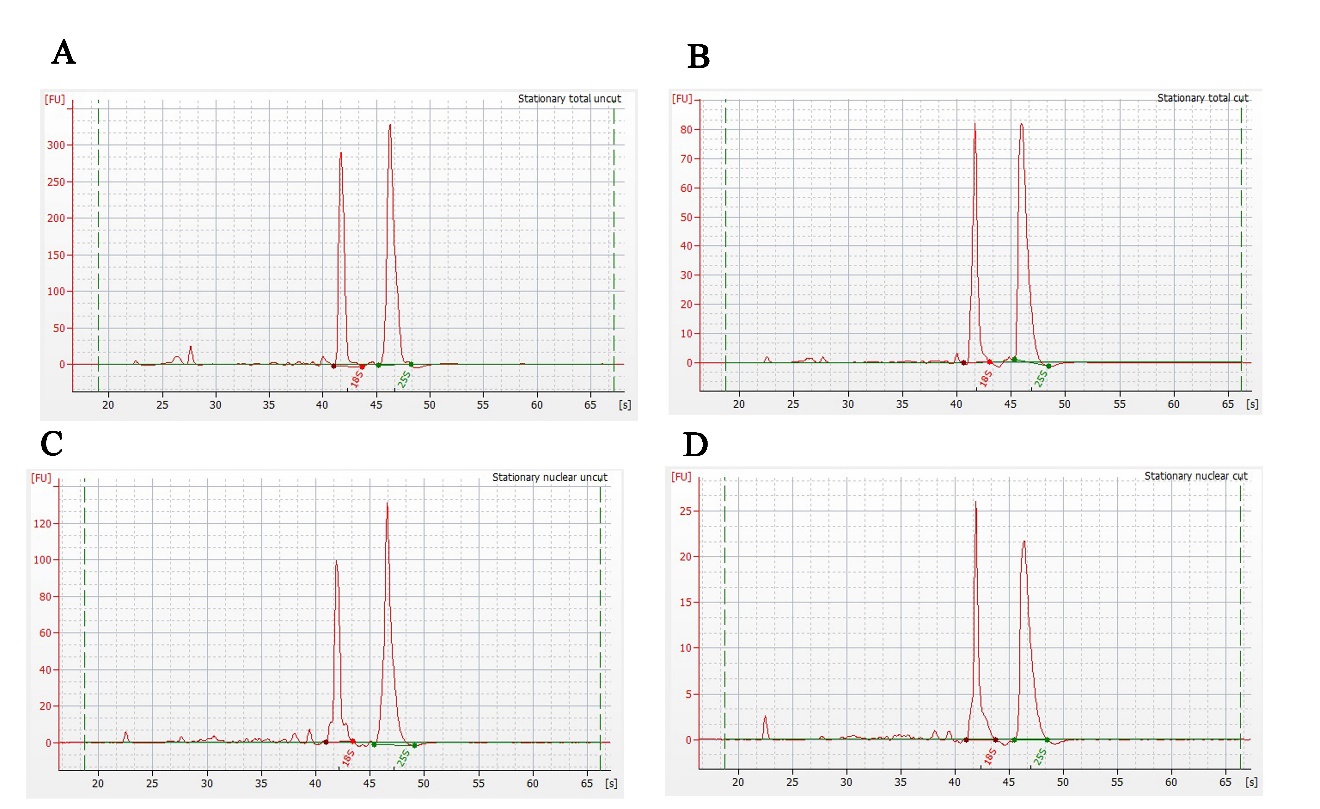


**Fig S2**. Representative electropherograms used to calculate Terminator resistance percentages in 18S and 25S. The areas under each peak were obtained using the Bioanalyzer Expert software. These areas were used to calculate the percentage of RNA resistance by obtaining the ratio between cut (Terminator treated) and uncut (untreated) RNA under different conditions. **a** total RNA from stationary organisms untreated and **b** treated with Terminator. **c** nuclear RNA from stationary organisms untreated and **d** treated with Terminator.


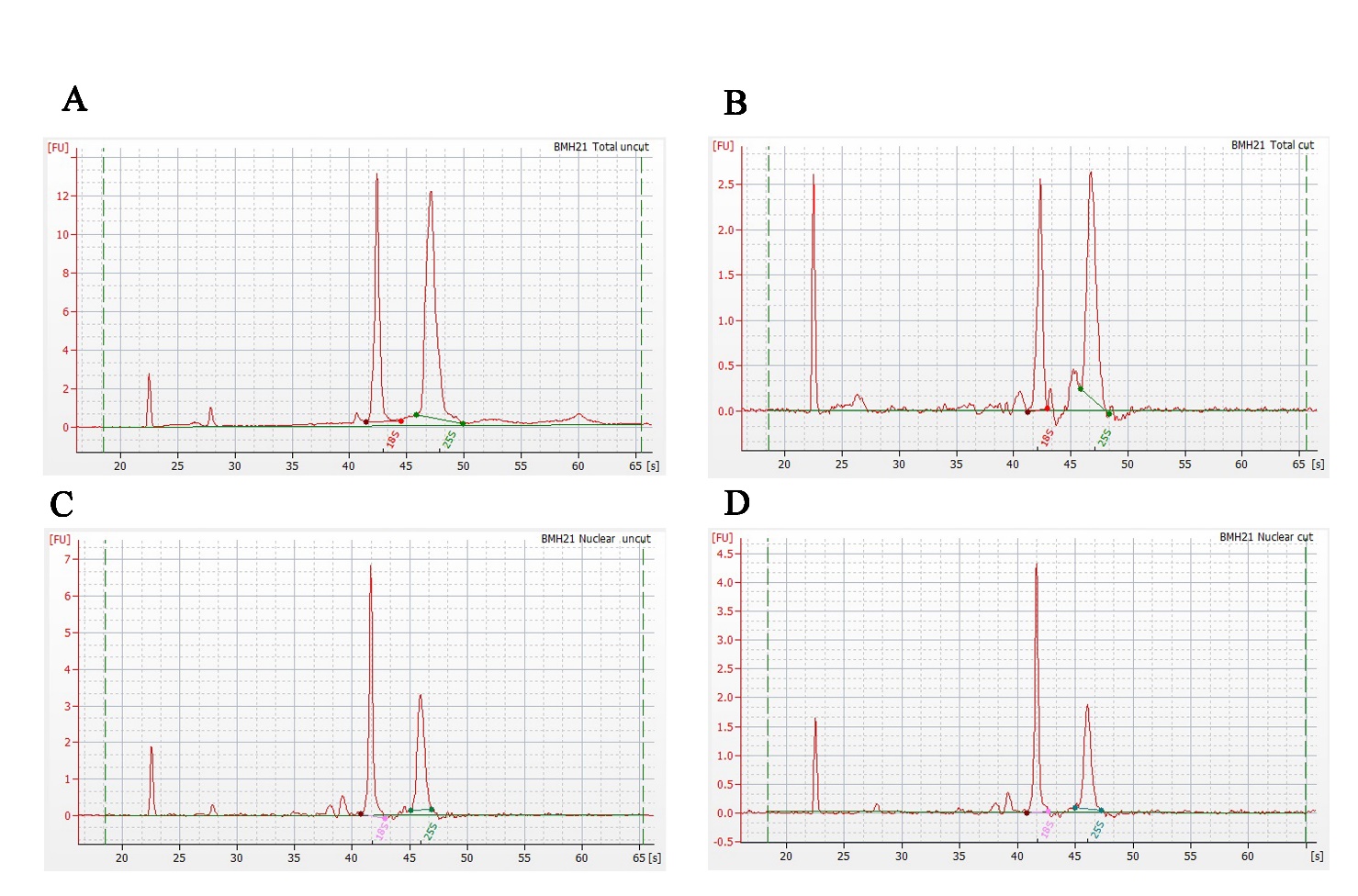


**Fig S3**. Representative electropherograms used to calculate Terminator resistance percentages in 18S and 25S. The areas under each peak were obtained using the Bioanalyzer Expert software. These areas were used to calculate the percentage of RNA resistance by obtaining the ratio between cut (Terminator treated) and uncut (untreated) RNA under different conditions. **a** total RNA from BMH-21 exposed organisms untreated and **b** treated with Terminator. **c** nuclear RNA from BMH-21 exposed organisms untreated and **d** treated with Terminator.


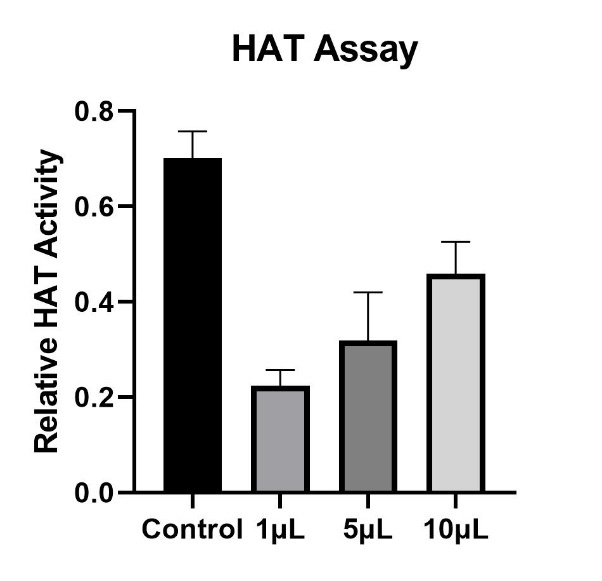


**Fig S4.** Histone Acetyltransferase Activity Assay (HAT) results. Nuclear extracts from *C. albicans* were compared at three different concentrations to a positive extract (control). HAT assays were carried out in order to verify that the nuclear RNA source was indeed the nucleus.


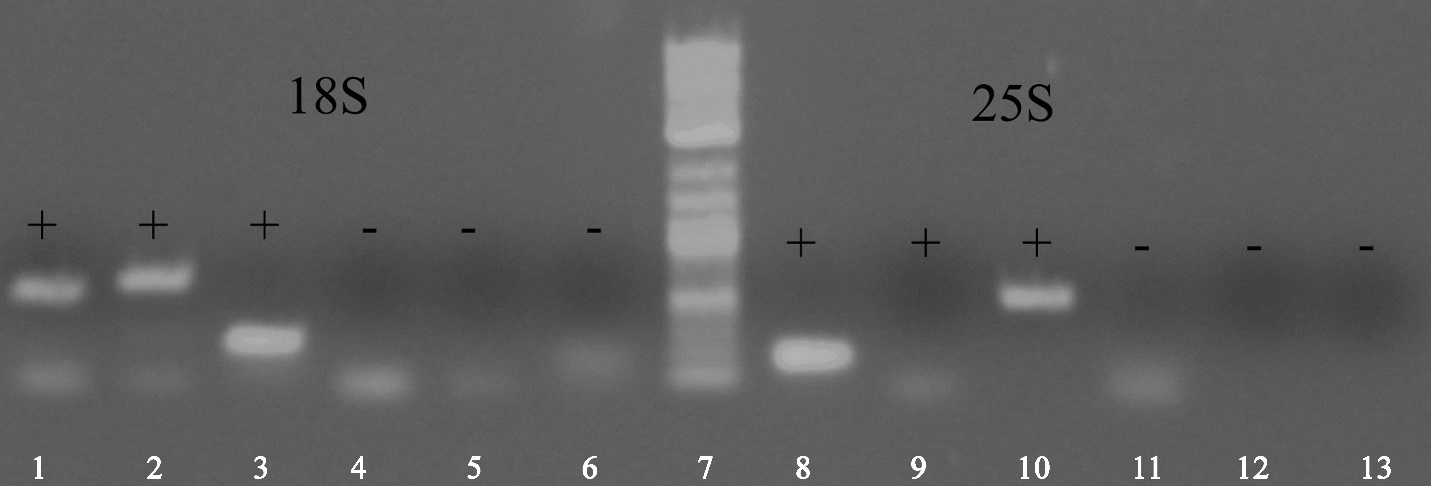


**Fig. S5.** Evidence for the role of RNA Pol II in the transcription of 18S and 25S molecules in stationary C. *albicans.* Chromatin Immunoprecipitation (ChIP) with polymerase II specific antibody. PCR fragments amplified from stationary organisms. Cells were cross-linked and chromatin was sheared by sonification. RNA Polymerase II mAb CTD4H8 (Epigentek) was used to precipitate DNA-protein complex. PCR was performed using three different sets of specific primers for 18S (PO-PB, PA-PP, PK-PQ) and 25S (PR-PD, PC-PS, PL-PT) (+). See Supporting Table 1 for primers information. A non-immune IgG antibody was used as negative control (-).

**TABLE S1.** List of primers used in all the experiments

| Primer designation | Primer Name | Sequence (5’-3’) | Experiment |
| --- | --- | --- | --- |
| PA | 18SfwdqPCR | AACGGCTACCACATCCAAGG | RT-qPCR, ChIP |
| PB | 18SrevqPCR | CACCAGACTTGCCCTCCAAT | RT-qPCR, ChIP, start sites |
| PC | 25SfwdqPCR | CAGGGATTGCCTCAGTAGCG | RT-qPCR, ChIP |
| PD | 25srevqPCR | CCCTCTGTGACGTCCTGTTC | RT-qPCR, ChIP, start sites |
| PE | ITS-1 fwd | AGCTGATTTGCTTAATTGCACCAC | RT-qPCR |
| PF | ITS-1 Rev | GACTATTAGTAATAATCTGGTGTGAC | RT-qPCR |
| PG | ITS-2 Fwd | GTCGTTTCTCCCTCAAACCGCT | RT-qPCR |
| PH | ITS-2 Rev | GTTTGAAGATATACGTGGTGGACGTT | RT-qPCR |
| PI | 5’-ETS-Fwd | CATCACGACCTACTTTATACGCTACG | RT-qPCR |
| PJ | 5’-ETS-Rev | CATCACGACCTACTTTATACGCTACG | RT-qPCR |
| P1 | 5.8S_Fwd | ACGGATCTCTTGGTTCTCGCATCGATGA | RT-qPCR |
| P2 | 5.8S Rev | AAACAGGCATGCCCTCCGGAATAC | RT-qPCR |
|  |  |  |  |
|  |  |  |  |
| PK | 18sFwd(b) | CTGGGGATAGAGCATTGTAATTGTT | PCR, ChIP |
| PL | 25S-3’ Fwd | GCAGTCAAGCGTTCATAGCG | PCR, ChIP |
| PM | P3mr-25S | GTCGCTGGACCATAGCAGGCTGGCAACG | PCR |
| PN | P1mr-18S | TCGATGGAAGTTTGAGGCAATAACAGGTCTGTG | PCR |
| PO | 18SFwd(a) | GCCAGTAGTCATATGCTTGTC | ChIP |
| PP | 18SREV_9 | ACCGATCCCTAGTCGGCATA | ChIP |
| PQ | 18S-3’_Rev_end | ATCCTTCCGCAGGTTCA | ChIP |
| PR | 25SFwd(a) | ATCAGGTAGGACTACCCGCTG | ChIP |
| PS | 25SREV_2 | GCCATAAGACCCCATCTCCG | ChIP |
| PT | 25S-3’_Rev_end | AATCAGACAACAAGAGCTTAA | ChIP |
| PU | GeneRacer Oligo | CGACUGGAGCACGAGGACACUGACAUGGACUGAAGGAGUAGAAA | Start sites |
| PV | GeneRacer 5’ | CGACTGGAGCACGAGGACACTGA | Start sites |
| PW | 25S-5’_Rev_Start | TCCAAACCGATGCTGG | Start sites |
| PX | 18S-5’_Rev_Start | AGCATGTATTAGCTCTAGAATTACC | Start sites |
| PY | 25S_rev_short | GGCAATCCCTGTTGGTTTCTT | Start sites |
| PZ | 18S_rev_short | CGCAGTTTCACTGTATAAATTGCTTATACTT | Start sites |
